# Supplementary material for: Use of Coronary CT Angiography to Predict Obstructive Lesions in Patients with Chest Pain without Enzyme and ST-Segment Elevation
Source: J Clin Med. 2021 Nov 21;10(22):5442. doi: 10.3390/jcm10225442 (PMC8625085; doi:10.3390/jcm10225442)
Supplement: Supplementary file 1 [file jcm-10-05442-s001.zip › jcm-1454666-supplementary.pdf]

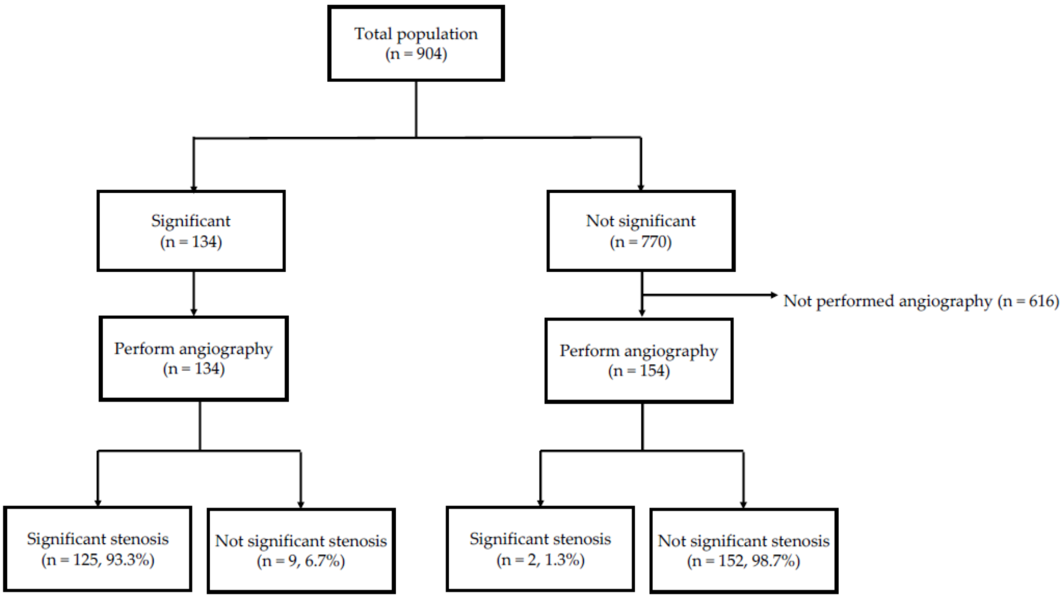

Figure S1. The result of coronary angiography according to presence of significant stenosis on CCTA.
